# Latent profile analysis of clinical nurses’ reflective ability in relation to dual work stress

**DOI:** 10.1371/journal.pone.0332077

**Published:** 2025-09-11

**Authors:** Jingru Song, Junxian Wu, Jing Yu, Lin Li, Mingfang Zhang, Qin Shen

**Affiliations:** 1 School of Nursing, Zhejiang Chinese Medical University, Hangzhou, China; 2 Liver and gallbladder surgery, Jiaxing First Hospital, Jiaxing, China; Jouf University, SAUDI ARABIA

## Abstract

**Objective:**

The aim of this study is to identify the latent profiles of reflective ability in clinical nurses and examine their association with dual work stress.

**Methods:**

A cross-sectional survey was conducted using convenience sampling between October 2023 and January 2024. Clinical nurses from three tertiary general hospitals in Jiaxing, Zhejiang Province, were recruited for the study. Data were collected using a general information questionnaire, the Clinical Nurses’ Reflective Capacity Scale, and the Dual Work Stress Scale. Latent profile analysis (LPA) was used to identify distinct reflective capacity profiles. Univariate analysis and logistic regression were employed to examine influencing factors.

**Results:**

A total of 843 nurses participated in the study, with a mean reflective capacity score of 56.15 ± 19.39. Four latent profiles were identified: Low-Level Negative Type (65.0%), High-Recall Insightful Type (7.0%), Low-Recall Introspective Type (4.0%), and High-Level Balanced Type (24.0%). Logistic regression analysis showed that challenge stress, hindrance stress, gender, professional title, participation in reflection training, the habit of keeping a reflective journal, and job satisfaction were significant factors influencing the latent profiles of reflective capacity (*P* < 0.05).

**Conclusion:**

Reflective capacity among clinical nurses exhibits heterogeneity, with four distinct latent profiles identified. Nursing managers should tailor interventions based on the influencing factors of different profiles to enhance reflective capacity in clinical nurses.

## Introduction

With the continuous advancement of public health awareness and the diversification of healthcare demands, patients have developed heightened expectations for both the technical proficiency and humanistic care in nursing services [1–3]. Concurrently, the increasingly complex clinical environment requires nurses to possess not only solid professional knowledge and skills but also the adaptability to manage emergent situations effectively. These include responding to acute clinical deteriorations, addressing patient emotional distress, and navigating ethical dilemmas [4]. To meet these challenges and continuously improve service quality, reflective ability has emerged as a pivotal competency in clinical practice [4]. Defined as a nurse’s capacity to consciously review clinical experiences, comprehend their cognitive and behavioral patterns, and derive practical wisdom from practice [5], reflective ability enables thorough self-evaluation across technical procedures, patient communication, and emotional support. This critical self-assessment ensures alignment with professional ethical standards and fosters ongoing professional development [6,7].

In clinical nursing practice, work stress significantly influences the development and application of clinical nurses’ reflective ability [8]. Work stress exhibits dual attributes, primarily comprising challenge stress and hindrance stress. Challenge stress refers to demanding yet growth-stimulating stressors, such as high job responsibility, excessive workload, and time pressure. In contrast, hindrance stress denotes stressors that are difficult to cope with and negatively impact work behavior, including role ambiguity and panic, lack of job security, restricted career development, and organizational policy constraints [9]. While fulfilling clinical duties, nurses must also undertake research tasks and meet promotion-related evaluation requirements, further increasing their workload and accelerating work pace. Additionally, factors such as frequent night shifts, fast-paced work environments, mismatched compensation and workload, and routine high-frequency assessments and inspections exacerbate nurses’ perception of hindrance stress while diminishing their positive appraisal of challenge stress [10].

To better understand the mechanism underlying the relationship between dual work stress and reflective ability among nurses, this study introduces the Job Demands-Resources (JD-R) model as the theoretical framework. This model divides the work environment into two categories: job demands and job resources. Job demands include high-intensity tasks and emotional load, which, when exceeding an individual’s coping capacity, can lead to burnout; job resources, on the other hand, include autonomy, organizational support, and psychological resources, which help buffer stress and facilitate goal achievement [11]. Dual work stress encompasses challenge stress and hindrance stress. From this theoretical perspective, while both types of stress fall under the category of job demands, challenge stress enhances professional growth by triggering achievement motivation, whereas hindrance stress has a negative impact by limiting developmental potential. Reflective ability, as a core psychological resource for nurses, helps them identify the attributes of stressors when coping with multiple pressures. Nurses with high reflective ability are better able to transform challenge stress into positive motivation and effectively alleviate the negative impact of hindrance stress, demonstrating significant adaptive advantages. In the context of the normalization of dual work stress in the healthcare industry, the cultivation of reflective ability should be regarded as a key intervention target for improving nursing quality and promoting sustainable professional development.

Although previous studies have validated the impact of dual work stress (Dual Work Stress) on the reflective ability (reflective ability) of clinical nurses, most past research has treated nurses as a homogeneous group and assessed their reflective ability using overall scores, without adequately considering the heterogeneity of clinical nurse populations in terms of demographic factors such as social demographics [10,12–14]. Traditional “one-size-fits-all” analytical approaches fail to reveal the potential characteristics of reflective ability among nurses from different backgrounds, thus limiting the specificity and effectiveness of training strategies. Some studies have pointed out that the current methods of cultivating reflective ability in clinical nurses are vague and lack targeted content [14], highlighting the urgent need to explore more specific pathways for improvement from the perspective of internal resource differences among nurses. Latent profile analysis (LPA) can categorize individuals based on differences in manifest items, so that the differences within each category are minimized while maximizing differences between categories [15]. Therefore, this study aims to investigate the current status of clinical nurses’ reflective ability, while introducing the concept of latent profile analysis (LPA) to more precisely identify the heterogeneous characteristics of clinical nurses’ reflective ability and analyze its influencing factors. Furthermore, it will explore the influence mechanism of dual work stress on the categorization of reflective ability types based on the JD-R theory. This research seeks to provide a theoretical basis for the development of personalized intervention strategies to improve clinical nurses’ reflective ability, enhance nursing quality, and promote the professional development of clinical nurses.

## Methods

### Objective

This study aims to investigate the current status of reflective ability in clinical nurses, using latent profile analysis (LPA) to identify different categories of reflective ability and explore their influencing factors. Additionally, it examines the association between dual work stress and reflective ability profiles in clinical nurses.

### Participants

Due to practical limitations (such as time, resource availability, and participant accessibility), this study recruited clinical nurses from three tertiary general hospitals in Jiaxing City, Zhejiang Province, using a convenience sampling method between October 2023 and January 2024. Although the convenience sampling method enhances the efficiency of data collection, it may introduce selection bias, as participants may not be representative of the broader population [16]. Furthermore, this study was conducted in three tertiary hospitals in Jiaxing City, Zhejiang Province. While these hospitals serve a diverse patient population, the geographic homogeneity may limit the direct applicability of our findings to other settings.Inclusion criteria: ①Currently employed and registered clinical nurses holding a valid nurse practice certificate; ②Fully informed and voluntarily consented to participate in this study. Exclusion criteria: ①Nurses absent from their posts during the survey period; ②Interns or nurses undergoing external training at other institutions.

### Sample size calculation

In October 2023, a preliminary survey was conducted with 30 clinical nurses using convenience sampling, revealing a standard deviation (*σ*) of 22.29 for the total reflective ability score. The sample size was calculated using the formula for cross-sectional studies: N = [Z*α*/2*σ/*δ*]^2^ [16], where Z*α*/2 = 1.96 (two-tailed *α* = 0.05) and the margin of error (*δ*) was set at 2. The initial calculation yielded a required sample size of N = 477. Accounting for a potential 20% attrition rate, the minimum sample size was determined to be N = 572. The study ultimately enrolled 843 clinical nurses, exceeding the minimum requirement. The research protocol was approved by the Medical Ethics Committee (Approval No. 2023-LY-427).

### Measures

#### General information questionnaire.

This questionnaire was self-developed by the researchers and includes 17 items covering the following demographic and professional characteristics: gender, age, years of work experience, marital status, education level, professional title, employment type, average monthly income, hospital grade, night shift frequency, average weekly overtime hours, department management role, teaching role, plans for further education, participation in training related to reflective ability, practice of keeping a reflection journal, and job satisfaction.

#### Reflective ability scale for clinical nurses.

This study employed the Reflective ability scale for clinical nurses, originally developed by Nishimoto et al. [5] in 2021 and subsequently translated into Chinese by Shao Lijiao et al. [17] in 2023, to assess nurses’ reflective competence. The scale comprises three dimensions: Recall of Nursing Practice, Reflection on Nursing Practice, and Expansion of Nursing Practice, encompassing a total of 19 items. Utilizing a 6-point Likert scale ranging from “strongly disagree” (1) to “strongly agree” (6), the total score ranges from 19 to 114, with higher scores indicating greater reflective ability. The reflective ability level was determined by calculating the score index, which represents the ratio of the actual score to the maximum possible score multiplied by 100%. A cutoff value of 70% was established, with scores below this threshold indicating a need for improvement in reflective ability. The original version of the scale exhibited the following construct validity indices: GFI = 0.914, AGFI = 0.890, CFI = 0.949, and RMSEA = 0.070, with a Cronbach’s *α* coefficient of 0.962. The Cronbach’s *α* coefficients for the three dimensions were 0.922, 0.918, and 0.885, respectively. For the Chinese version of the scale, content validity was evaluated with an S-CVI of 0.947 and an I-CVI range of 0.857 to 1.000. The construct validity fit indices for the Chinese version were RMSEA = 0.052, CFI = 0.941, GFI = 0.902, and IFI = 0.942. The overall Cronbach’s *α* coefficient was 0.948, with the Cronbach’s *α* coefficients for the three dimensions being 0.869, 0.703, and 0.794, respectively.

#### Challenge and Hindrance-Related Self-Reported Stress Scale.

This study utilized the Challenge and Hindrance-Related Self-Reported Stress Scale, originally developed by Cavanaugh et al [9]. in 2000 and subsequently translated, culturally adapted, and revised by Zhang Yi et al [18]. in 2013, to assess dual work stress among clinical nurses. The scale consists of two subscales: Challenge Stress (6 items) and Hindrance Stress (5 items), comprising a total of 11 items. A 5-point Likert scale was employed, ranging from “no stress at all” (1) to “extremely high stress” (5), with higher scores indicating greater perceived stress levels. The construct validity indices for the original version of the scale were as follows: CFI = 0.90, NNFI = 0.87, and Cronbach’s *α* coefficients for the two subscales were 0.87 and 0.75, respectively. For the Chinese version of the scale, the construct validity indices were GFI = 0.80, NFI = 0.85, CFI = 0.86, and RMSEA = 0.09. The overall Cronbach’s *α* coefficient for the scale was 0.925, while the Cronbach’s *α* coefficients for the two subscales were 0.919 and 0.875, respectively.

### Data collection

The survey was conducted anonymously using Questionnaire Star platform(https://www.wjx.cn/). Prior to distribution, approval was obtained from the relevant departments within the participating institutions. A link to the questionnaire was provided to nurses in the hospital, along with an explanation of the survey’s purpose, significance, and instructions for completing the questionnaire. Each respondent was limited to a single submission, and all questions were mandatory; only fully completed questionnaires could be successfully submitted. After data collection, questionnaires were screened, and invalid responses were excluded based on the following criteria: ①Answers exhibited obvious patterns or identical responses across all items; ②Responses were logically inconsistent; ③Completion time was less than 5 minutes. A total of 900 questionnaires were distributed, of which 843 were valid, resulting in an effective response rate of 93.67%.

### Dtatistical method

The dataset of this study consists of continuous observational variables, and thus latent profile analysis (LPA) was used to identify different types of reflective ability. Broadly speaking, latent profile analysis is a modeling technique that categorizes individuals into distinct subgroups based on differences in the data [19]. Compared to traditional methods such as analysis of variance (ANOVA) and regression analysis, the advantage of latent profile analysis lies in its ability to more precisely understand the similarities between individuals and how differences between them are captured and identified. This is because classification is not solely based on observed characteristics (e.g., gender and age) [16]. Latent profile analysis also outperforms traditional clustering analysis [20], specifically in the following ways: ①greater flexibility in model estimation; ②higher accuracy in classification; ③the ability to include predictor variables to enhance the ability to differentiate between categories.

### Data analysis

Statistical analysis was performed using Mplus 8.3 to fit latent profiles of nurses’ reflective ability. The analysis started with a one-class initial model and progressively increased the number of latent classes. The optimal model was determined based on evaluation indices, goodness-of-fit tests, and difference tests. Commonly used model fit indices included the Akaike Information Criterion (AIC), Bayesian Information Criterion (BIC), and adjusted BIC (aBIC), with smaller values indicating better model fit. Entropy was used as an indicator of classification precision, ranging from 0 to 1, with values closer to 1 reflecting higher precision. Additionally, the Lo-Mendell-Rubin adjusted likelihood ratio test (LMR) and the Bootstrap-based likelihood ratio test (LMRT) were employed to verify differences in model fit. The interpretability and practical significance of each latent class were also considered in selecting the optimal model. Subsequent data analysis was conducted using SPSS 26.0. Continuous variables were expressed as mean ± standard deviation, and group comparisons were performed using one-way analysis of variance (ANOVA). Categorical variables were expressed as frequencies and percentages, with group comparisons conducted using chi-square tests or rank-sum tests. Variables with statistical significance in univariate analysis were included as independent variables, while latent profiles served as the dependent variables in unordered multinomial logistic regression analysis. A two-tailed significance level of *α* = 0.05 was used, with *P* < 0.05 considered statistically significant.

### Ethical statement

This study was approved by the Medical Ethics Committee (Approval No. 2023-LY-427) and adheres to the principles outlined in the Declaration of Helsinki. All participants were adults over the age of 18 and provided written informed consent prior to their involvement in the study. The confidentiality of participants was strictly maintained, and all survey data were anonymized, being used solely for research purposes, with access restricted to authorized personnel only. The research team complied with both national and international ethical standards and guidelines throughout the entire study process. All participants were explicitly informed prior to the study that the questionnaire might contain sensitive questions related to work stress, with clear instructions that they could skip any questions or withdraw from the study at any time without penalty. The three participating hospitals each maintained dedicated psychological support teams to provide necessary counseling for nurses who might experience distress during the pressure and reflective ability assessments. Furthermore, the study employed the Wenjuanxing online platform for anonymous data collection, utilizing coded IDs instead of real names to prevent identification by supervisory staff, thereby reducing social desirability bias and potential psychological stress associated with questionnaire completion

## Results

### Reflective ability and dual work stress levels of clinical nurses

The reflective ability score of the 843 clinical nurses was (56.15 ± 19.39), the Challenge Stress score was (17.10 ± 6.32), and the Hindrance Stress score was (16.65 ± 5.50).

### Latent profiles analysis and naming of reflective ability among clinical nurses

Using the 19 item scores from the Clinical Nurses’ Reflective Ability Scale as observed indicators, we estimated latent profile models ranging from 1 to 5 classes (see Table 1). After comprehensive evaluation, the four-profile solution was identified as optimal based on the following criteria [21]: ①An entropy value of 0.89, demonstrating excellent classification accuracy; ②Significant BLRT results (*p* < 0.001) favoring the 4-class solution over the 3-class alternative; ③Distinct elbow points in both AIC and BIC values at the 4-class solution; ④Complete theoretical coherence across all derived profiles. Although the 5-class solution yielded marginally superior AIC values, it included one profile comprising only 3% of participants, thereby violating established minimum class size requirements.

**Table 1:** Fitting information of potential profile model of reflective ability of clinical nurses.

Model	Loglikelihood	AIC	BIC	aBIC	Entropy	LMR	BLRT	Class Probability
1	−28634.369	57344.738	57524.743	57404.067	/	/	/	1.00
2	−25535.914	51187.827	51462.571	51278.382	0.980	<0.001	<0.001	0.69/0.31
3	−24573.939	49303.878	49673.362	49425.658	0.987	0.001	0.001	0.07/0.65/0.28
4	−23650.961	47497.922	47962.145	47650.928	0.994	<0.001	<0.001	0.65/0.07/0.04/0.24
5	−22785.347	45806.695	46365.657	45990.926	0.959	0.014	0.015	0.17/0.22/0.34/0.11/0.16

### Naming the latent profiles of clinical nurses’ reflective ability

**Fig 1:**
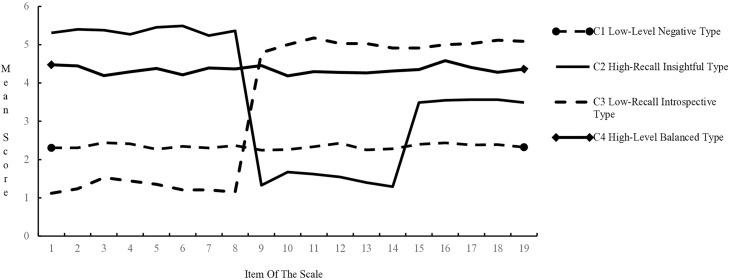
Mean scores for each item of the four-profile model of clinical nurses’ reflective ability (n = 843). The X-axis represents the 19 items of the Reflective Ability Scale for Clinical Nurses. The Y-axis represents the mean score for each item.

Taking Model 4 as the ideal model, clinical nurses’ reflective ability can be divided into four categories: Clinical nurses in the first category (n = 550, 65.0%) scored between 2 and 3 points on the average of all items, and scored at a low level in all dimensions. At the same time, nurses in this category had low satisfaction with their work content, but the low participation rate of reflective training and reflection diary recording rate reflected their lack of enthusiasm to improve the current situation. Hence the name “Low-Level Negative Type”; Clinical nurses in the second category (n = 55, 7.0%) scored the highest in “Recall Their Own Nursing Practice”, reflecting their careful observation of nursing process and insight in recalling details of nursing practice. Hence the name “High-Recall Insightful Type”; Clinical nurses in the third category (n = 34, 4.0%) scored lower on “Recall Their Own Nursing Practice”, The scores of “Reflect on Their Own Nursing Practice” and “Expand Their Own Nursing Practice” are both high, indicating that although there are fewer retrospective behaviors, But it is good at reflecting and reconstructing nursing experience, so it is named “Low-Recall Introspective Type”; The clinical nurses in the fourth category (n = 204, 24.0%) scored between 4 and 5 points on the average of each item, which was at a High Level and Balanced in the three dimensions, so they were named “high-level Balanced Type” (See Fig 1).

### Univariate analysis of latent profiles of reflective ability among clinical nurses

Univariate analysis revealed statistically significant differences (*P* < 0.05) in the distribution of nurses across different reflective ability profiles based on gender, years of work experience, professional title, participation in reflection-related training, habit of keeping reflective journals, job satisfaction, Challenge Stress scores, and Hindrance Stress scores (see Tables 2 and 3).

**Table 2:** General characteristics of participants and univariate analysis of latent profiles of reflective ability.

	Overall Clinical Nurses	Low-Level Negative Type (n = 550)	High-Recall Insightful Type (n = 55)	Low-Recall Introspective Type (n = 34)	High-Level Balanced Type (n = 204)	*F*	*P*
Challenge Stress	17.10 ± 6.32	16.17 ± 6.02	16.75 ± 8.33	16.88 ± 5.88	19.76 ± 5.83	17.059	<0.001
Hindrance Stress	16.65 ± 5.50	17.59 ± 4.89	18.40 ± 6.02	20.35 ± 6.94	13.04 ± 4.97	48.613	<0.001

**Table 3:** Comparison of challenge and hindrance stress scores across different latent profiles (n = 843, Mean ± SD).

	Low-Level Negative Type (n = 550)	High-Recall Insightful Type (n = 55)	Low-Recall Introspective Type (n = 34)	High-Level Balanced Type (n = 204)	*χ* ^*2*^ */H*	*P*
Gender					18.162	0.001
Male	57 (10.4%)	7 (12.7%)	9 (26.5%)	42 (20.6%)		
Female	493 (89.6%)	48 (87.3%)	25 (73.5%)	162 (79.4%)		
Age (years)					2.450	0.484
≤25	93 (16.9%)	9 (16.4%)	6 (17.6%)	25 (12.3%)		
26 ~ 30	204 (37.1%)	20 (36.4%)	13 (38.2%)	78 (38.2%)		
31 ~ 40	204 (37.1%)	22 (40.0%)	14 (41.1%)	79 (38.7%)		
≥41	49 (8.9%)	4 (7.3%)	1 (2.9%)	22 (10.8%)		
Years of Working (year)					23.224	<0.001
≤5	232 (42.2%)	25 (45.5%)	4 (11.8%)	62 (30.4%)		
6 ~ 10	162 (29.5%)	19 (34.5%)	16 (47.1%)	59 (28.9%)		
11 ~ 20	130 (23.6%)	10 (18.2%)	10 (29.4%)	66 (32.4%)		
≥21	26 (4.7%)	1 (1.8%)	4 (11.8%)	17 (8.3%)		
Degree of Education					0.559	0.906
Associate degree or above	110 (20.0%)	14 (25.5%)	8 (23.5%)	53 (26.0%)		
Bachelor degree	415 (75.5%)	38 (69.1%)	24 (70.6%)	130 (63.7%)		
Master degree or above	25 (4.5%)	3 (5.5%)	2 (5.9%)	21 (10.3%)		
Professional Title					43.533	<0.001
Nurse	207 (37.6%)	22 (40.0%)	4 (11.8%)	45 (22.1%)		
Nurse practitioner	191 (34.7%)	15 (27.3%)	5 (14.7%)	60 (29.4%)		
Nurse-in-charge	131 (23.8%)	17 (30.9%)	21 (61.8%)	84 (41.2%)		
Associate chief nurse and above	21 (3.8%)	1 (1.8%)	4 (11.8%)	15 (7.4%)		
Whether Participated in Reflection-Related Training					79.181	<0.001
Yes	237 (43.1%)	41 (74.5%)	24 (70.6%)	155 (76.0%)		
No	313 (56.9%)	14 (25.5%)	10 (29.4%)	49 (24.0%)		
Whether Kept Reflective Journals					60.238	<0.001
Yes	201 (36.5%)	36 (65.5%)	21 (61.8%)	132 (64.7%)		
No	349 (63.5%)	19 (34.5%)	13 (38.2%)	72 (35.3%)		
Level of Satisfaction with Nursing Work					108.786	<0.001
Very disgruntled	119 (21.6%)	15 (27.3%)	6 (17.6%)	9 (4.4%)		
Dissatisfied	181 (32.9%)	17 (30.9%)	2 (5.9%)	43 (21.1%)		
Ordinary	203 (36.9%)	10 (18.2%)	12 (35.3%)	66 (32.4%)		
Satisfied	25 (4.5%)	7 (12.7%)	5 (14.7%)	25 (12.3%)		
Quite satisfied	22 (4.0%)	6 (10.9%)	9 (26.5%)	61 (29.9%)		

### Multivariate analysis of latent profiles of reflective ability among clinical nurses

Variables with statistical significance in univariate analysis were included as independent variables, while the latent profiles of nurses’ reflective ability were treated as the dependent variable in an unordered multinomial logistic regression analysis. Collinearity diagnostics showed that the tolerance values of the constructed model ranged from 0.786 to 0.968, and variance inflation factors (VIF) were between 1.033 and 1.272, indicating no multicollinearity among the independent variables [22](see Table 5). The results showed that gender, professional title, participation in reflection-related training, habit of keeping reflective journals, job satisfaction, Challenge Stress, and Hindrance Stress were significant factors influencing the latent profiles of nurses’ reflective ability (*P* < 0.05) (see Table 4).

**Table 4:** Influencing factors of latent profile of reflective ability of clinical nurses.

	Low-Level Negative Type (n = 550)				High-Recall Insightful Type (n = 55)				Low-Recall Introspective Type (n = 34)			
	*B*	*P*	*OR*	*95%CI*	*B*	*P*	*OR*	*95%CI*	*B*	*P*	*OR*	*95%CI*
Gender (female as reference)												
Male	−0.561	0.041	0.571	0.333 ~ 0.977	−0.599	0.199	0.549	0.220 ~ 1.369	0.576	0.247	1.780	0.671 ~ 4.718
Professional title (nurse as reference)												
Nurse practitioner	−0.757	0.004	0.469	0.280 ~ 0.787	−0.750	0.072	0.472	0.209 ~ 1.071	0.255	0.734	1.290	0.297 ~ 5.603
Nurse-in-charge	−1.606	<0.001	0.201	0.119 ~ 0.338	−0.926	0.022	0.396	0.180 ~ 0.873	1.244	0.051	3.470	0.992 ~ 12.138
Associate chief nurse and above	−1.703	<0.001	0.182	0.076 ~ 0.436	−1.846	0.092	0.158	0.018 ~ 1.351	2.145	0.016	8.539	1.502 ~ 48.558
Have you participated in training related to reflection (based on participation in reflection training)												
No	−1.304	<0.001	0.271	0.175 ~ 0.422	−0.302	0.434	0.739	0.347 ~ 1.576	−0.589	0.227	0.555	0.213 ~ 1.443
Whether to keep a reflection diary (refer to keeping a reflection diary)												
No	−0.791	<0.001	0.453	0.298 ~ 0.690	0.060	0.867	1.062	0.527 ~ 2.141	0.107	0.817	1.113	0.449 ~ 2.757
Work satisfaction (with dissatisfaction as reference)												
Dissatisfied	−0.151	0.610	0.860	0.481 ~ 1.537	−0.077	0.866	0.926	0.379 ~ 2.263	−1.677	0.066	0.187	0.031 ~ 1.117
Ordinary	−0.053	0.857	0.949	0.536 ~ 1.680	−0.617	0.216	0.540	0.203 ~ 1.435	0.260	0.681	1.297	0.375 ~ 4.489
Satisfied	−0.942	0.021	0.390	0.175 ~ 0.869	−0.050	0.930	0.952	0.312 ~ 2.898	0.523	0.490	1.687	0.382 ~ 7.444
Quite satisfied	−1.321	0.001	0.267	0.125 ~ 0.568	−0.540	0.348	0.583	0.189 ~ 1.799	1.123	0.106	3.074	0.787 ~ 12.001
Challenge Stress	−0.047	0.007	0.954	0.922 ~ 0.987	−0.003	0.913	0.997	0.943 ~ 1.054	0.019	0.619	1.020	0.945 ~ 1.100
Hindrance Stress	0.128	<0.001	1.137	1.093 ~ 1.182	0.171	<0.001	1.187	1.111 ~ 1.268	0.303	<0.001	1.353	1.227 ~ 1.492

**Table 5:** Test for multicollinearity.

Model	Unstandardized Coefficients		Standardized Coefficients			Collinearity Statistics	
	*B*	*SE*	Beta	*t*	*P*	*Tolerance*	*VIF*
Constant	3.924	.313		12.543	.000		
Gender	−.222	.112	−.060	−1.979	.048	.968	1.033
Professional title	−.297	.048	−.198	−6.145	.000	.840	1.191
Have you participated in training related to reflection	−.417	.086	−.162	−4.851	.000	.786	1.272
Whether to keep a reflection diary	−.238	.086	−.093	−2.779	.006	.790	1.266
Work satisfaction	.118	.035	.106	3.404	.001	.899	1.112
Challenge Stress	.025	.007	.122	3.741	.000	.821	1.219
Hindrance Stress	−.051	.008	−.216	−6.588	.000	.815	1.227

## Discussion

### Latent profiles of reflective ability among clinical nurses: Identification and distribution

The total reflective ability score among clinical nurses was 56.1 ± 19.39, significantly lower than the score (89.93) reported by Zhao et al. [12] for specialist nurses. This discrepancy may stem from fundamental differences in their professional roles and career trajectories. Specialist nurses typically engage in longitudinal practice within specialized domains, facilitating more systematic and in-depth reflection. In contrast, clinical nurses manage diverse patient populations with fragmented workflows, lacking opportunities for focused practice and profound reflection, which may constrain reflective capacity development.

Although previous studies [12–14] have established the critical importance of reflective ability for nursing quality, most assessments relied solely on total or dimensional scores, failing to uncover latent heterogeneity within the nursing population. For instance, while Wu et al. [23] and Long et al. [13] examined reflective patterns across nurse types, neither identified potential reflective typologies, potentially obscuring individual differences. Our latent profile analysis classified clinical nurses’ reflective ability into four distinct profiles: ①’Low-Level Negative Type’, ②’High-Recall Insightful Type’, ③’Low-Recall Introspective’, and ④’High-Level Balanced Type’. These profiles demonstrated significant differences in both total and dimensional scores (all *p* < 0.001), confirming substantial population heterogeneity. Notably, the ‘Low-level Negative’ profile predominated (65.0%), while the ‘High-level Balanced’ profile represented 24.0% of the sample.“

### Demographic and professional characteristics associated with reflective profiles

Gender plays a significant role in the types of reflective ability. Male nurses are less likely to be categorized into the “Low-Level Negative Type” than female nurses (*OR* = 0.571, *P* < 0.05), which may be related to the greater family and childcare responsibilities typically shouldered by female nurses, limiting their time and energy for deep reflection [24]. Furthermore, nurses in the “Low-Level Negative Type” have significantly lower job satisfaction than those in the “High-Level Balanced Type” (*OR* = 0.267, *P* < 0.05). This result is inconsistent with some previous studies [25], and a possible explanation is that the majority of nurses in this category have an associate degree. With the inclusion of nursing programs in China under “National Control” management [26], the educational threshold has been continuously raised. Clinical nurses are facing increased pressure regarding professional title advancement and performance evaluations, especially for those with lower educational levels or newly hired nurses, who must cope with heavy clinical workloads alongside the demands of further education. The imbalance between professional rewards and efforts is more likely to trigger psychological gaps and job burnout, which in turn weakens their professional identity and reflective motivation. Therefore, nursing managers should pay particular attention to the structural vulnerabilities exhibited by nurses in the “Low-Level Negative Type,” especially in terms of education level, years of employment, and access to resources. For this group, it is essential to enhance guided reflective training, moderately reduce workload pressures, and provide development-oriented incentives to effectively improve their reflective motivation and job satisfaction.

Nurses in the “High-Recall Insightful Type” exhibit relatively low overall job satisfaction, but they demonstrate a higher frequency of reflective behavior. This study found that nurses in this category had higher participation rates in reflective training and greater frequency of using reflective journals compared to other types, indicating strong problem awareness and reflective motivation. This seemingly contradictory phenomenon may stem from their higher level of self-awareness [27], a finding that has been validated in one study. Additionally, research has shown that reflective insight is positively correlated with humanistic care abilities (*r* = 0.275, *P* < 0.01) [28]. This suggests that nursing managers should recognize the potential for humanistic care in this group of nurses and may enhance their empathy through narrative reflective training. Previous studies have also pointed out that a decrease in job satisfaction may drive individuals to engage in more frequent reviews of situations, problem analysis, and experience accumulation, with emotional distress often being a key trigger for deep reflection [29]. Therefore, in promoting the development of reflective abilities, nursing managers should focus on supporting and guiding “High-Recall Insightful Type” nurses to transform their reflective behaviors from problem-driven to growth-oriented. Given their low job satisfaction but strong reflective willingness, structured reflective guidance, emotional support mechanisms, and case supervision could be used to help them effectively internalize reflection as a positive driving force for professional growth, thereby avoiding negative emotional rumination and enhancing their professional adaptability and job satisfaction [30].

Nurses in the “Low-Recall Introspective Type” are predominantly from the high-ranking professional group. While they possess a certain level of self-awareness, their reflective behavior is mainly characterized by unidirectional individual introspection. This may be related to the lack of retrospective triggers and supportive feedback loops for these nurses, which limits both the depth and breadth of their reflection. This has been validated in previous studies [31]. Additionally, some research suggests that high-ranking nurses, who are often clinically experienced and skilled in operations, tend to rely on experiential judgment for quick decision-making. This, in turn, weakens their need for systematic reviews of work situations and peer feedback [32]. Specifically, high-ranking nurses, due to their reduced involvement in routine basic nursing tasks, may base their reflection on individual impressions rather than peer comparisons. Their reflective outcomes also lack clear output pathways or action validation, leading to a “reflection without expression” and “cognition without transformation” state of reflective stagnation. Over time, this could not only limit the depth of their professional growth but also hinder their potential to play a leadership role in reflective practice. However, the small sample size of high-ranking nurses in this study may result in an unstable estimation of this group’s reflective characteristics. Furthermore, the limitations in sampling methods and regional selection in this study may restrict the generalizability of the findings. Additionally, unmeasured potential confounding factors such as managerial roles and years of specialized work experience may also affect the interpretation of the results. Future research could validate the phenomenon of reflective stagnation among high-ranking nurses through longitudinal studies.

### The role of dual work pressure in shaping reflective ability

Dual work stress has a significant impact on reflective ability types. The higher the challenge stress, the more likely nurses are to be categorized into the “High-Level Balanced Type” (*OR* = 0.954, *P* < 0.05); conversely, the higher the hindrance stress, the more likely they are to be categorized into the “Low-Level Negative Type” (*OR* = 0.128, *P* < 0.001). In comparison, nurses in the “High-Level Balanced Type” exhibit stronger resource mobilization and reflective integration abilities, enabling them to more effectively transform challenge stress into a driving force for professional growth. According to the Job Demands–Resources (JD-R) model, an individual’s cognitive perception of work demands and their level of mobilizable resources jointly determine their coping pathways and behavioral responses [33]. Although challenge stress is accompanied by task demands, it is often viewed as a stimulating factor with development potential, capable of fostering nurses’ initiative and learning motivation. With reflective ability and resource support, nurses can transform challenges into growth drivers, enhancing problem-solving and professional judgment through reflection, thereby creating a positive “stress-reflection-growth” cycle [34]. In contrast, when a systematic reflective mechanism or organizational support is lacking, nurses are more likely to perceive work demands as hindrance stress, leading to negative emotions and cognitive overload. Reflective behaviors may become superficial or even avoided, ultimately limiting their professional development [32]. Therefore, nursing managers should focus on early interventions for reflective mechanisms, helping nurses establish positive stress coping patterns and reflective habits through continuous training, situational guidance, and resource support, thereby enhancing their professional resilience and capacity for sustainable development.

## Conclusion

This study, through latent profile analysis, is the first to systematically identify four heterogeneous types of reflective ability among clinical nurses, with the “Low-Level Negative Type” accounting for the highest proportion (65.0%), reflecting the structural reflective dilemma currently faced by the clinical nursing population. The primary finding of the study is that dual work stress has a differentiated impact on reflective ability, with challenge stress promoting professional growth, while hindrance stress exacerbates reflective obstacles. Additionally, nurses with different demographic characteristics exhibit significant reflective differences; female and low-education nurses are more likely to fall into negative reflective patterns, while high-ranking nurses exhibit “reflective stagnation,” where their unidirectional introspection may limit the depth of professional development. The use of convenience sampling in this study may result in insufficient sample representativeness or an overestimation of the overall reflective ability level. Furthermore, the data was only sourced from three tertiary hospitals in the Yangtze River Delta region, excluding nurses from Western regions and grassroots medical institutions. Additionally, the small sample size of high-ranking nurses, with only 15 samples (3.2%) of associate chief nurses and above, leads to an overly wide confidence interval for their odds ratio (*OR*), so the conclusions should be interpreted with caution. Variables such as managerial roles and years of specialized work experience were not included in the analysis, which may also influence the estimation of the stress-reflection relationship. Future studies could improve the methodology by using stratified random sampling, proportionally allocating by professional title and hospital grade, and expanding data collection to multi-center studies in Central and Western regions. Longitudinal tracking could also be conducted to verify the dynamic formation mechanism of “reflective stagnation” in high-ranking nurses. Moreover, mixed research methods, such as interviews, could be employed to explore the organizational cultural factors underlying the different reflective types.

### Limitations

This study has several limitations that should be acknowledged. First, the cross-sectional design precludes causal inferences regarding the relationship between reflective ability and work stress. Longitudinal or intervention studies are needed to establish temporal relationships and assess changes in reflection patterns over time. Second, the study has sampling limitations. Convenience sampling was used, and participants were recruited from three tertiary hospitals in a single region (Jiaxing), which may restrict the generalizability of the findings to other healthcare settings (e.g., primary care or rural hospitals). Additionally, the small subgroup size—particularly the underrepresentation of high-ranking nurses (e.g., associate chief nurses and above)—resulted in wide confidence intervals for odds ratio estimates and reduced statistical power for this subgroup. Furthermore, reflective ability and work stress were assessed using self-reported questionnaires, which may be subject to social desirability bias or recall inaccuracies. Future studies could incorporate observational or peer-assessment methods to validate the findings. Moreover, potential confounding variables—such as workload intensity, shift patterns, or organizational culture—were not accounted for in the analysis. These factors may influence both reflective ability and perceived stress levels. Finally, this study was conducted within the Chinese healthcare system, where nursing roles and workplace dynamics may differ from those in other countries. Caution should be exercised when generalizing the results to international contexts.
